# Supplementary material for: Modelling Skylarks (Alauda arvensis) to Predict Impacts of Changes in Land Management and Policy: Development and Testing of an Agent-Based Model
Source: PLoS One. 2013 Jun 6;8(6):e65803. doi: 10.1371/journal.pone.0065803 (PMC3675089; doi:10.1371/journal.pone.0065803)
Supplement: Supporting Information S4 — The skylark ODdox as a zipped archive. (ZIP) [file pone.0065803.s004.zip › Skylark_ODdox/functions_vars_0x6d.html]

ALMaSS Skylark ODdox: Class Members - Variables


ALMaSS Skylark ODdox
 2.0


- Main Page
- Related Pages
- Classes
- Files

- Class List
- Class Index
- Class Hierarchy
- Class Members

- All
- Functions
- Variables
- Typedefs

- a
- b
- c
- d
- e
- f
- g
- h
- i
- l
- m
- n
- o
- p
- r
- s
- t
- v
- x
- y

### - m -

- m\_Age
  : RoeDeerInfo
- m\_agrochemindustrycereal
  : Farm
- m\_ALandscape
  : MovementMap16
  , PositionMap
  , MovementMap
- m\_AlleleFreqsFile
  : Population\_Manager
- m\_amount
  : PesticideEvent
- m\_area
  : LE
- m\_aTerrlist
  : Skylark\_Adult
- m\_baddays
  : Skylark\_Clutch
- m\_BaitList
  : RodenticideManager
- m\_BLtype
  : BaitLocation
- m\_bool
  : CfgBool
- m\_border
  : LE
- m\_Born\_x
  : Skylark\_Base
- m\_Born\_y
  : Skylark\_Base
- m\_BreedingAttempts
  : Skylark\_Female
- m\_BreedingSuccess
  : Skylark\_Female
- m\_Brood
  : Skylark\_Male
- m\_BroodSize
  : Skylark\_Male
- m\_bug\_percent\_a
  : CropData
- m\_bug\_percent\_b
  : CropData
- m\_bug\_percent\_c
  : CropData
- m\_bug\_percent\_d
  : CropData
- m\_cangrow
  : Edges
- m\_carrots
  : Farm
- m\_catastrophestartyear
  : Population\_Manager
- m\_cattle\_grazing
  : LE
- m\_centre\_x
  : EdgeGrowingPoints
- m\_centre\_y
  : EdgeGrowingPoints
- m\_centroidx
  : LE
- m\_centroidy
  : LE
- m\_CGG1
  : Farm
- m\_CGG2
  : Farm
- m\_cgridcount
  : Population\_Manager
- m\_cgridcountwidth
  : Population\_Manager
- m\_CIPEGrid
  : Skylark\_Female
- m\_cipegridsize
  : Population\_Manager
- m\_colourRes
  : BinaryMapBase
- m\_colourScaler
  : BinaryMapBase
- m\_competitionscaler
  : skTTerritory
- m\_corr\_x
  : Pesticide
- m\_corr\_y
  : Pesticide
- m\_count
  : Crop
- m\_Counter1
  : Skylark\_Female
- m\_countrydesignation
  : LE
- m\_CurrentSkState
  : Skylark\_Base
- m\_curve\_num
  : VegElement
- m\_cx
  : EGP\_Data
- m\_cy
  : EGP\_Data
- m\_Dad
  : Skylark\_Nestling
- m\_daily\_spray\_queue
  : Pesticide
- m\_date
  : Calendar
- m\_DateCut
  : RoadsideVerge
  , Orchard
  , OrchardGrass
- m\_datemodulus
  : Weather
- m\_day\_in\_month
  : Calendar
- m\_day\_in\_year
  : Calendar
- m\_daylength
  : Calendar
- m\_days\_since\_insecticide\_spray
  : LE
- m\_ddegs
  : LE
- m\_dds
  : CropGrowth
- m\_dead\_biomass
  : VegElement
- m\_deathRate
  : RodenticideManager
- m\_diffusion\_mask
  : Pesticide
- m\_diffusionRate
  : RodenticideManager
- m\_digestability
  : VegElement
- m\_dx
  : Diffusor
- m\_dy
  : Diffusor
- m\_EasyPopRes
  : Population\_Manager
- m\_EggNumber
  : Skylark\_Female
- m\_element
  : LowPriPair< ELEMTYPE >
- m\_elems
  : Landscape
- m\_EleType
  : AnimalPosition
- m\_EM
  : Skylark\_Nestling
- m\_EM\_fail
  : Skylark\_Nestling
- m\_ev
  : Crop
- m\_event
  : FarmEvent
- m\_farm
  : Crop
- m\_farm\_num
  : Farm
- m\_farmfunc\_tried\_to\_do
  : LE
- m\_FarmManager
  : Landscape
- m\_farmmapping
  : Landscape
- m\_farms
  : FarmManager
- m\_farmtype
  : Farm
- m\_field
  : FarmEvent
  , Crop
- m\_fieldpeas
  : Farm
- m\_fieldpeasstrigling
  : Farm
- m\_fields
  : Farm
- m\_file\_type
  : LE
- m\_FileRecord
  : probe\_data
- m\_first\_date
  : Crop
- m\_first\_year
  : FarmEvent
- m\_firstPF
  : Skylark\_Male
- m\_firstyear
  : Calendar
- m\_float
  : CfgFloat
- m\_fodderbeet
  : Farm
- m\_foddergrass
  : Farm
- m\_for\_iter\_x
  : SkTerritories
- m\_for\_iter\_y
  : SkTerritories
- m\_force\_growth
  : VegElement
- m\_force\_LAgreen
  : VegElement
- m\_force\_LAtotal
  : VegElement
- m\_force\_veg\_height
  : VegElement
- m\_force\_Weed
  : VegElement
- m\_forced\_phase\_shift
  : VegElement
- m\_fraction
  : Diffusor
- m\_GeneticsFile
  : Population\_Manager
- m\_green\_biomass
  : VegElement
- m\_gridcount
  : Population\_Manager
- m\_gridcountsize
  : Population\_Manager
- m\_gridcountwidth
  : Population\_Manager
- m\_gridsize
  : RodenticideManager
- m\_GrNeed
  : Skylark\_Nestling
- m\_growth
  : CropData
- m\_HabitatTable\_PNum
  : Skylark\_Male
- m\_HabitatTable\_Size
  : Skylark\_Male
- m\_hash\_size
  : SkTerritories
- m\_height
  : Landscape
  , RasterMap
  , RodenticideManager
  , BinaryMapBase
- m\_height10
  : Landscape
- m\_heightm1
  : RodenticideManager
- m\_herbicidedelay
  : LE
- m\_heterogeneity
  : skTTerritory
- m\_high
  : LE
- m\_hours
  : Calendar
- m\_hr\_polys
  : skTTerritory
- m\_hr\_sizes
  : skTTerritory
- m\_id
  : RasterMap
- m\_ifile
  : VegElement
  , CropData
- m\_immigrationRate
  : RodenticideManager
- m\_insect\_pop
  : VegElement
- m\_InsectTable
  : Skylark\_Male
- m\_insolation
  : Weather
- m\_int
  : CfgInt
- m\_intensity
  : Farm
- m\_is\_in\_map
  : LE
- m\_IsBadWeather
  : Skylark\_Population\_Manager
- m\_janfirst
  : Calendar
- m\_key
  : CfgBase
- m\_LAgreen
  : VegElement
- m\_land
  : Landscape
  , Pesticide
  , RodenticideManager
- m\_largeroad\_load
  : LE
- m\_LargestPolyNumUsed
  : Landscape
- m\_last\_date
  : Crop
- m\_lastindex
  : LE
- m\_LastSprayed
  : OrchardBand
- m\_lasttreat
  : LE
- m\_lastyear
  : Calendar
- m\_LAtotal
  : VegElement
- m\_level
  : CfgBase
  , MapErrorMsg
- m\_lineno
  : Configurator
- m\_ListNameLength
  : Population\_Manager
- m\_ListNames
  : Population\_Manager
- m\_Location\_x
  : TAnimal
  , skTTerritory
- m\_Location\_y
  : TAnimal
  , skTTerritory
- m\_lock
  : FarmEvent
- m\_lownut
  : CropGrowth
- m\_MainForm
  : Population\_Manager
- m\_maize
  : Farm
- m\_maizesilage
  : Farm
- m\_maizestrigling
  : Farm
- m\_management\_loop\_detect\_count
  : LE
- m\_management\_loop\_detect\_date
  : LE
- m\_map
  : Pesticide
  , RasterMap
  , BinaryMapBase
- m\_map\_index
  : LE
- m\_maplength
  : BinaryMapBase
- m\_marchfirst
  : Calendar
- m\_mask
  : BinaryMapBase
- m\_maskbits
  : BinaryMapBase
- m\_mass
  : BaitLocation
- m\_maxx
  : LE
  , SimplePositionMap
  , PositionMap
- m\_maxy
  : LE
  , SimplePositionMap
  , PositionMap
- M\_Mig\_Mort
  : Skylark\_Population\_Manager
- m\_MinFemaleAcceptScore
  : Skylark\_Female
- m\_minutes
  : Calendar
- m\_minx
  : LE
- m\_miny
  : LE
- m\_month
  : Calendar
- m\_monthly\_traffic
  : LE
- m\_mowndecay
  : LE
- m\_MyFile
  : probe\_data
- m\_MyFileName
  : probe\_data
- m\_MyHome
  : Skylark\_Base
- m\_MyMinTerritoryQual
  : Skylark\_Male
- m\_nest\_pos\_validx
  : skTTerritory
- m\_nest\_pos\_validy
  : skTTerritory
- m\_nest\_valid
  : skTTerritory
- m\_NestLeavingChance
  : Skylark\_Nestling
- m\_NestTime
  : Skylark\_Female
- m\_newgrowthsum
  : VegElement
- m\_newoldgrowthindex
  : VegElement
- m\_next\_tov
  : FarmEvent
- m\_NoAreas
  : probe\_data
- m\_NoChickDeaths
  : Skylark\_Population\_Manager
- m\_NoEleTypes
  : probe\_data
- m\_NoFarms
  : probe\_data
- m\_NoFledgeDeaths
  : Skylark\_Population\_Manager
- m\_NoPestEffects
  : Skylark\_Population\_Manager
- m\_NoProbes
  : Population\_Manager
- m\_NoVegTypes
  : probe\_data
- m\_num\_crops
  : CropData
- m\_numbers
  : CropData
- m\_nutrient\_status
  : VegElement
- m\_oats
  : Farm
- m\_OBarleyPCG
  : Farm
- m\_ocarrots
  : Farm
- m\_OCGG1
  : Farm
- m\_OCGG2
  : Farm
- m\_OCGS1
  : Farm
- m\_ofieldpeas
  : Farm
- m\_ofieldpeassilage
  : Farm
- m\_ofirstyeardanger
  : Farm
- m\_ograzingpigs
  : Farm
- m\_olddays
  : Calendar
  , LE
- m\_oldLAtotal
  : VegElement
- m\_oldnewgrowth
  : VegElement
- m\_OldRange\_x
  : RoeDeerInfo
- m\_OldRange\_y
  : RoeDeerInfo
- m\_omaizesilage
  : Farm
- m\_ooats
  : Farm
- m\_opermgrassgrazed
  : Farm
- m\_opotatoes
  : Farm
- m\_orig\_size
  : EGP\_Data
- m\_originalsize
  : EdgeGrowingPoints
- m\_osbarleysilage
  : Farm
- m\_ospringbarley
  : Farm
- m\_ospringbarleyext
  : Farm
- m\_ospringbarleypigs
  : Farm
- m\_OurEdges
  : EdgeGrowingPoints
- m\_OurLandscape
  : TAnimal
- m\_OurPopulationManager
  : Skylark\_Base
- m\_OurTerritories
  : Skylark\_Base
- m\_owinterbarley
  : Farm
- m\_owinterbarleyext
  : Farm
- m\_owinterrape
  : Farm
- m\_owinterrye
  : Farm
- m\_owinterwheatundersown
  : Farm
- m\_owinterwheatundersownext
  : Farm
- m\_owner
  : LE
- m\_owner\_file
  : LE
- m\_owner\_index
  : LE
- m\_pcide\_conc
  : Skylark\_Base
- m\_permanentsetaside
  : Farm
- m\_PermCrops
  : Farm
- m\_permgrassgrazed
  : Farm
- m\_permgrasslowyield
  : Farm
- m\_permgrasstussocky
  : Farm
- m\_pest\_daily\_decay\_frac
  : Pesticide
- m\_pest\_map\_height
  : Pesticide
- m\_pest\_map\_main
  : Pesticide
- m\_pest\_map\_mask
  : Pesticide
- m\_pest\_map\_size
  : Pesticide
- m\_pest\_map\_twin
  : Pesticide
- m\_pest\_map\_width
  : Pesticide
- m\_pesticide\_accumulation
  : Skylark\_Base
- m\_pesticide\_sprayed
  : Skylark\_Female
  , Skylark\_Male
- m\_pesticide\_sprayed\_die
  : Skylark\_Female
- m\_PesticideGridCell
  : LE
- m\_PesticideType
  : Landscape
- m\_pig\_grazing
  : LE
- m\_pmouse\_map\_main
  : RodenticideManager
- m\_pmouse\_map\_twin
  : RodenticideManager
- m\_poison
  : LE
- m\_poly
  : LE
- m\_poly\_seen
  : SkTerritories
- m\_poly\_size
  : SkTerritories
- m\_polylist
  : PolygonDataVector
- m\_polynum
  : EdgeGrowingPoints
  , EGP\_Data
- m\_polyrefs
  : PolygonDataVector
- m\_polys
  : skTTerritory
  , SkQualGrid
- m\_potatoes
  : Farm
- m\_potatoesindustry
  : Farm
- m\_pri
  : LowPriPair< ELEMTYPE >
- m\_prop
  : Pesticide
- m\_qual
  : SkQualGrid
- m\_qual\_cache
  : SkTerritories
- m\_qual\_cache\_filled
  : SkTerritories
- m\_qual\_grid
  : SkTerritories
- m\_qual\_grid\_signal
  : SkTerritories
- m\_Quality
  : skTTerritory
- m\_queue
  : Farm
- m\_rain
  : Weather
- m\_rainingtoday
  : Weather
- m\_raintoday
  : Weather
- m\_range\_div10
  : skTTerritory
- m\_Range\_x
  : RoeDeerInfo
- m\_Range\_y
  : RoeDeerInfo
- m\_Rect
  : probe\_data
- m\_RefEle
  : probe\_data
- m\_RefFarms
  : probe\_data
- m\_RefVeg
  : probe\_data
- m\_repeat\_start
  : LE
- m\_ReportInterval
  : probe\_data
- m\_resolution
  : BinaryMapBase
- m\_resolutionscaler
  : BinaryMapBase
- m\_RodenticideManager
  : Landscape
- m\_rot\_index
  : LE
- m\_rotation
  : Farm
- m\_rotation\_sync\_index
  : Farm
- m\_rots
  : CropRotation
- m\_run
  : FarmEvent
- m\_running
  : LE
- m\_sbarleyclovergrass
  : Farm
- m\_ScaleFactor
  : ScalablePositionMap
- m\_seasonstartprob
  : RodenticideManager
- m\_seedgrass1
  : Farm
- m\_seedgrass2
  : Farm
- m\_setaside
  : Farm
- m\_signal\_mask
  : LE
- m\_sim\_h\_div\_10
  : SkTerritories
- m\_sim\_w\_div\_10
  : SkTerritories
- m\_SimulationName
  : Population\_Manager
- m\_simulationyear
  : Calendar
- m\_Size
  : Skylark\_Base
  , RoeDeerInfo
- m\_sizes
  : SkQualGrid
  , skTTerritory
- m\_skylarkscrapes
  : LE
- m\_slopes
  : CropGrowth
- m\_smallroad\_load
  : LE
- m\_snow\_decay
  : Weather
- m\_snowtoday
  : Weather
- m\_soiltype
  : LE
- m\_something\_to\_decay
  : Pesticide
- m\_sprayed\_elem
  : PesticideEvent
- m\_springbarley
  : Farm
- m\_springbarleyclovergrassstrigling
  : Farm
- m\_springbarleypeaclovergrassstrigling
  : Farm
- m\_springbarleyptreatment
  : Farm
- m\_springbarleyseed
  : Farm
- m\_springbarleysilage
  : Farm
- m\_springbarleyskmanagement
  : Farm
- m\_springbarleystrigling
  : Farm
- m\_springbarleystriglingculm
  : Farm
- m\_springbarleystriglingsingle
  : Farm
- m\_springrape
  : Farm
- m\_squares\_in\_map
  : LE
- m\_start
  : CropGrowth
  , CropRotation
- m\_start\_valid
  : CropGrowth
- m\_startday
  : FarmEvent
- m\_startDay
  : BaitLocation
- m\_StepSize
  : Population\_Manager
- m\_stockfarmer
  : Farm
- m\_StriglingMort
  : Skylark\_Population\_Manager
- m\_string
  : CfgStr
- m\_subtype
  : LE
- m\_TargetTypes
  : probe\_data
- m\_temp
  : Weather
- m\_temptoday
  : Weather
- m\_TheBitMaskArray
  : PositionMap
- m\_TheBitMaskArray2
  : PositionMap
- m\_TheLandscape
  : Population\_Manager
- m\_TheMap
  : MovementMap16
  , SimplePositionMap
  , MovementMap
  , PositionMap
  , IDMap
- m\_Time
  : probe\_data
- m\_todayslength
  : Calendar
- m\_todo
  : FarmEvent
- m\_toowet
  : Skylark\_Female
- m\_TotalEggs
  : Skylark\_Population\_Manager
- m\_TotalNestlings
  : Skylark\_Population\_Manager
- m\_TotalPrefledgelings
  : Skylark\_Population\_Manager
- m\_toxShouldSpray
  : Landscape
- m\_tramlinesdecay
  : LE
- m\_treatment\_counts
  : Landscape
- m\_tried\_to\_do
  : LE
- m\_triticale
  : Farm
- m\_type
  : LE
- m\_unsprayedmarginpolyref
  : LE
- m\_user
  : LE
- m\_useThisYear
  : BaitLocation
- m\_valid\_x
  : LE
- m\_valid\_y
  : LE
- m\_veg\_biomass
  : VegElement
- m\_veg\_cover
  : VegElement
- m\_veg\_density
  : VegElement
- m\_veg\_height
  : VegElement
- m\_veg\_patchy
  : VegElement
- m\_veg\_phase
  : VegElement
- m\_vegage
  : LE
- m\_vege\_danger\_store
  : LE
- m\_vege\_type
  : VegElement
- m\_VegType
  : AnimalPosition
- m\_versioninfo
  : Landscape
- m\_VirtualDiameter
  : skTTerritory
- m\_warnfile
  : MapErrorMsg
- m\_weed\_biomass
  : VegElement
- m\_weed\_curve\_num
  : VegElement
- m\_weed\_percent
  : CropData
- m\_width
  : Landscape
  , BinaryMapBase
  , RodenticideManager
  , RasterMap
- m\_width10
  : Landscape
- m\_wind
  : Weather
- m\_wind\_valid
  : Weather
- m\_winddir
  : Weather
- m\_winddir\_valid
  : Weather
- m\_winddirtoday
  : Weather
- m\_windtoday
  : Weather
- m\_winterbarley
  : Farm
- m\_winterbarleystrigling
  : Farm
- m\_winterrape
  : Farm
- m\_winterrapestrigling
  : Farm
- m\_winterrye
  : Farm
- m\_winterryestrigling
  : Farm
- m\_winterwheat
  : Farm
- m\_winterwheatstrigling
  : Farm
- m\_winterwheatstriglingculm
  : Farm
- m\_winterwheatstriglingsingle
  : Farm
- m\_wwheatpcontrol
  : Farm
- m\_wwheatptoxiccontrol
  : Farm
- m\_wwheatptreatment
  : Farm
- m\_x
  : Point
  , Edges
  , AnimalPosition
  , BaitLocation
  , APoint
  , RasterMap
- m\_x1
  : rectangle
- m\_x2
  : rectangle
- m\_x\_add
  : Landscape
- m\_x\_div10
  : skTTerritory
- m\_x\_excess
  : Pesticide
- m\_XFNestAcceptScore
  : Skylark\_Male
- m\_xmaxx
  : PositionMap
- m\_y
  : BaitLocation
  , AnimalPosition
  , Edges
  , RasterMap
  , APoint
  , Point
- m\_y1
  : rectangle
- m\_y2
  : rectangle
- m\_y\_add
  : Landscape
- m\_y\_div10
  : skTTerritory
- m\_y\_excess
  : Pesticide
- m\_yddegs
  : LE
- m\_year
  : Calendar
- m\_youngforest
  : Farm
- maxx
  : MovementMap16
  , IDMap
  , MovementMap
- maxy
  : MovementMap16
  , IDMap
  , MovementMap
- MConsts
  : LE
- MDates
  : LE
- mh
  : Skylark\_struct
- MinDegrees
  : Skylark\_Clutch
- month
  : aDate
- Mother
  : Skylark\_Clutch
- Mum
  : skClutch\_struct
- MyClutch
  : Skylark\_Female
- MyExtractEff
  : Skylark\_Adult
- MyMate
  : Skylark\_Female
  , Skylark\_Male
- MyTerritory
  : Skylark\_Adult


- Generated on Thu Jan 10 2013 13:15:39 for ALMaSS Skylark ODdox by
   1.8.1.1
